# Draft Genome Sequence of Stenotrophomonas maltophilia Strain PE591, a Polyethylene-Degrading Bacterium Isolated from Savanna Soil

**DOI:** 10.1128/MRA.00490-21

**Published:** 2021-08-12

**Authors:** Tayná Diniz Frederico, Julianna Peixoto, Jéssica Fernandes de Sousa, Carla Simone Vizzotto, Andrei Stecca Steindorff, Otávio Henrique Bezerra Pinto, Ricardo Henrique Krüger

**Affiliations:** a Enzymology Laboratory, Department of Cell Biology, University of Brasília, Brasília, Brazil; b Department of Civil and Environmental Engineering, University of Brasília, Brasília, Brazil; c U.S. Department of Energy Joint Genome Institute, Lawrence Berkeley National Laboratory, Berkeley, California, USA; Montana State University

## Abstract

We report the genome sequence of a polyethylene-degrading bacterial strain identified as Stenotrophomonas maltophilia strain PE591, which was isolated from plastic debris found in savanna soil. The genome was assembled in 16 scaffolds with a length of 4,751,236 bp, a GC content of 66.5%, and 4,432 predicted genes.

## ANNOUNCEMENT

Stenotrophomonas maltophilia strain PE591 was isolated from plastic debris found in the soil of the Brazilian Cerrado biome (1). The strain showed both metabolic activity and cellular viability after incubation with unpretreated polyethylene (PE) (molecular weight, 191,000) films as the sole carbon source for periods of up to 90 days (1). Moreover, S. maltophilia PE591 was capable of inducing significant physicochemical changes in PE after a 90-day incubation, revealing its great potential for plastic biodegradation processes (1). The *Stenotrophomonas* genus currently comprises 20 species, and its first species, S. maltophilia, was described in 1993 (2–4). S. maltophilia is a Gram-negative, obligate aerobic, rod-shaped, and motile bacterium that is considered to be an important human pathogen (5). *Stenotrophomonas* species are ubiquitous microorganisms that colonize multiple natural (e.g., soils and plants) and clinical environments (6, 7). In addition, *Stenotrophomonas* spp. may have resistance to different metals and antibiotics; therefore, they qualify as promising microorganisms for bioremediation applications (6).

S. maltophilia strain PE591 was grown in 5 ml of nutrient broth medium (Difco, Holland) and incubated for 24 h at 28°C with agitation (150 × *g*). Subsequently, cells were harvested by centrifugation at 5,500 × *g* for 5 min at 4°C. Bacterial DNA was purified using the GenElute bacterial genomic DNA kit (Sigma-Aldrich, USA). A sequencing library was created using the MiSeq reagent kit (Illumina, San Diego, CA) according to the manufacturer’s instructions, the MiSeq system user guide, revision L (part number 15027617; Illumina), was used in the sequencing protocol, and genome sequencing was performed with the Illumina MiSeq system (2 × 300-bp paired-end reads) (Macrogen, Seoul, South Korea). The resultant reads were subjected to quality analysis using FastQC software v. 0.11.3 (8). Sequence reads were *de novo* assembled following the A5-miseq pipeline, which includes Trimmomatic v. 0.35 to trim low-quality sequences (Phred scores of <20) and IDBA-UD v. 1.1.1 to assemble contigs (9–11). The package Stats from BBmap v. 38.76 was used to generate assembly statistics (12). The genome was annotated with Prokka v. 1.14.6 using the UniProt database (13). The completeness and contamination of genomic data were estimated by CheckM v. 1.0.13 (14). Coverage was assessed with SAMtools v. 1.9, Bowtie2 v. 2.3.4.1, and the package Pileup from BBmap v. 38.76 (12, 15, 16). Default parameters were used for all software unless otherwise noted. Finally, the taxonomic classification was assessed following the analysis of (i) the average nucleotide identity (ANI) obtained through the web server JSpeciesWS (http://jspecies.ribohost.com/jspeciesws/#analyse), which generates the ANIb (ANI algorithm using BLAST), and (ii) digital DNA-DNA hybridization (dDDH) obtained through the Type (Strain) Genome Server (TYGS) (https://tygs.dsmz.de) (17, 18).

The genome sequencing generated a total of 13,411,628 reads, with a total of 4,019,163,037 bp. The genome of S. maltophilia PE591 was assembled in 16 contigs, with a total length of 4.75 Mb and a GC content of 66.5%. Genome coverage, completeness, and contamination were ∼800×, 100%, and 0%, respectively. *N*_50_ and *L*_50_ values for the assembly were 473,413 bp and 3 contigs, respectively. Genomic functional features include a total of 4,432 genes, 4,350 protein-coding genes, 7 rRNAs, 74 tRNAs, and 1 transfer-messenger RNA.

The genomic ANI of S. maltophilia PE591 in relation to Stenotrophomonas maltophilia strain NCTC10258 (GenBank accession number NZ_LS483377.1) was 97.75% (ANIb). The dDDH (d4) value provided by the TYGS against Stenotrophomonas maltophilia NBRC 14161 (RefSeq assembly accession number GCF_001591205.1) was 80.2%. According to these results and the minimum parameters for taxonomic classification of prokaryotes using genomic data (19), strain PE591 is classified as a novel strain of Stenotrophomonas maltophilia. Unraveling the genomes of PE degraders contributes to efforts to find strategies to address the worldwide accumulation of persistent plastics. The potential of S. maltophilia PE591 to break and to oxidize the most recalcitrant plastic qualifies it as a promising biological tool for tackling this urgent global issue.

### Data availability.

This whole-genome shotgun project has been deposited in DDBJ/ENA/GenBank under the accession number JAAOKO000000000.1, with BioProject accession number PRJNA609181 (sample, *Stenotrophomonas* sp. strain PE591 [BioSample accession number SAMN14238446]; experiment, PE591 [SAR accession number SRX10931469]; run, PE_591_1.fastq.gz [SRA accession number SRR14584137]).
